# Shared Midgut Binding Sites for Cry1A.105, Cry1Aa, Cry1Ab, Cry1Ac and Cry1Fa Proteins from *Bacillus thuringiensis* in Two Important Corn Pests, *Ostrinia nubilalis* and *Spodoptera frugiperda*

**DOI:** 10.1371/journal.pone.0068164

**Published:** 2013-07-05

**Authors:** Carmen Sara Hernández-Rodríguez, Patricia Hernández-Martínez, Jeroen Van Rie, Baltasar Escriche, Juan Ferré

**Affiliations:** 1 Departamento de Genética, Universitat de València, Burjassot, Spain; 2 Bayer CropScience N.V., Ghent, Belgium; Ghent University, Belgium

## Abstract

First generation of insect-protected transgenic corn (Bt-corn) was based on the expression of Cry1Ab or Cry1Fa proteins. Currently, the trend is the combination of two or more genes expressing proteins that bind to different targets. In addition to broadening the spectrum of action, this strategy helps to delay the evolution of resistance in exposed insect populations. One of such examples is the combination of Cry1A.105 with Cry1Fa and Cry2Ab to control *O. nubilalis* and *S. frugiperda*. Cry1A.105 is a chimeric protein with domains I and II and the C-terminal half of the protein from Cry1Ac, and domain III almost identical to Cry1Fa. The aim of the present study was to determine whether the chimeric Cry1A.105 has shared binding sites either with Cry1A proteins, with Cry1Fa, or with both, in *O. nubilalis* and in *S. frugiperda*. Brush-border membrane vesicles (BBMV) from last instar larval midguts were used in competition binding assays with ^125^I-labeled Cry1A.105, Cry1Ab, and Cry1Fa, and unlabeled Cry1A.105, Cry1Aa, Cry1Ab, Cry1Ac, Cry1Fa, Cry2Ab and Cry2Ae. The results showed that Cry1A.105, Cry1Ab, Cry1Ac and Cry1Fa competed with high affinity for the same binding sites in both insect species. However, Cry2Ab and Cry2Ae did not compete for the binding sites of Cry1 proteins. Therefore, according to our results, the development of cross-resistance among Cry1Ab/Ac, Cry1A.105, and Cry1Fa proteins is possible in these two insect species if the alteration of shared binding sites occurs. Conversely, cross-resistance between these proteins and Cry2A proteins is very unlikely in such case.

## Introduction

The spraying of insecticidal products on corn plant surfaces is a strategy of limited effectiveness because the larvae from several species tunnel throughout the stem or feed from the roots. Genetically engineered corn plants expressing Cry proteins from *Bacillus thuringiensis* (Bt-corn) can effectively control stem borers, ear feeders and rootworms, reducing at the same time environmental costs associated with the use of conventional insecticides [1], [2]. Bt-corn was first commercially grown in 1996 and nowadays it is grown in many countries, occupying a global surface of 47.4 million hectares in 2011 [3].

The first cultivated Bt-corn expressed a single *B. thuringiensis* protein, Cry1Ab, which is highly active against the European corn borer, *Ostrinia nubilalis* (one of the most important lepidopteran pests in northern production areas). This was the only type of Bt-corn that was commercially planted until relatively recently and it accounts for most of the area planted worldwide. Other types of Bt-corn that have been developed later on are those designed for the control of rootworms, expressing Cry3 proteins (YieldGard VT®, Agrisure RW®) or binary Cry34/35 proteins (Herculex RW®). Bt-corn expressing Cry1Fa (Herculex I®) has been developed for Bt-corn cultivation in warmer areas in order to control *Spodoptera* spp, barely susceptible to Cry1Ab. Commercial agreements among companies have led to the stacking of these insecticidal proteins, conferring dual protection against above- and underground insect pests. Combination of several *cry* genes not only serves to broaden the protection against a higher number of insect pests, but it is also meant to delay the onset of resistance when more than one insecticidal protein is active against the same insect species [2], [4]–[7].

Several commercially available Bt-corn products contain the event MON 89034, which combines two lepidopteran active Cry proteins: Cry2Ab and Cry1A.105 (http://www.utcrops.com/corn/corn_insects/pubs_pdf/BtCornTraits.pdf, accessed 2013 Jun), [7]. Cry1A.105 is a chimeric protein with domains I and II and the C-terminal half of the protein from Cry1Ac, and domain III almost identical to Cry1Fa [8]. It is worth to note that Cry1Ab and Cry1Ac proteins are neighbors in the phylogenetic trees clustering domains I and II, and mainly differ in domain III [9]. Bt-corn expressing the combination of Cry2Ab and the Cry1A.105 means to provide protection to a wide range of highly destructive lepidopteran corn pests, including European corn borer (*O. nubilalis*), Southwestern corn borer (*Diatraea grandiosella*), Southern cornstalk borer (*Diatraea crambidoides*), corn earworm (*H. zea*), fall armyworm (*Spodoptera frugiperda*), corn stalk borer (*Papaipema nebris)*, and sugarcane borer (*Diatraea saccharalis).* More recently, for a more effective control of *Spodoptera* spp, new combinations of the event MON 89034 have led to novel Bt-crops expressing Cry1A.105, Cry2Ab and Cry1Fa [7], [10].

Since the very beginning of the Bt-crop technology, it was fully recognized that the main threat for the long-term success of such crops is the potential of insects to develop resistance [11], [12]. With the increase in the adoption of Bt-crops worldwide, the need for implementing resistance management strategies is impelling. In addition to the mandatory use of structured refuges in the U.S. (http://www.epa.gov/pesticides/biopesticides, accessed 2013 Jun) and in Australia, pyramiding Cry proteins is the strategy of choice. If the targets of two Cry proteins are different, the insect should carry two mutations to become resistant to both toxins. Therefore, this rationale is based on the occurrence of different targets for the proteins that are pyramided. If the two pyramided proteins share a binding site in the midgut of the larva, a single mutation altering such binding site could confer resistance to both proteins, making the resistance strategy useless [13]–[17].

The lack of cross-resistance between Cry1A and Cry2A proteins is well documented [2], [5], [16], and it has been shown that these proteins bind to different sites in several heliothine species [16], [18], [19]. Considering that the alteration of the binding to the insect midgut is the step of the mode of action that has most often been associated with insect resistance to Cry proteins [2], [5], the chances of cross resistance between Cry2Ab and Cry1A.105 should be low (although other resistance mechanisms cannot be discarded). However, a number of cases of cross-resistance to Cry1Fa in insects selected with Cry1A proteins have been documented [20]–[22]. Moreover, binding of Cry1A proteins and Cry1Fa to the same binding sites has been shown in several insect species [23]–[25], including *S. frugiperda* [26]. The aim of the present study was to determine whether the chimeric Cry1A.105 has shared binding sites either with Cry1A proteins, with Cry1Fa, or with both, in *O. nubilalis* and in *S. frugiperda*, two of the most damaging lepidopteran pests of corn in North and South America, respectively. Additionally, two Cry2A proteins were included in the study. Results from this paper could assess the potential of these two species, which have already been exposed to Cry1Ab or to Cry1Fa in the field, to develop cross-resistance to Cry1A.105 based on mutations altering common binding sites.

## Materials and Methods

### Insects

Eggs from *O. nubilalis* were obtained from the Institut National de Recherche Agronomique (INRA, Montpellier, France). Laboratory colonies of *O. nubilalis* and *S. frugiperda* were maintained in a climate chamber at 25.0±0.3°C, 70±5% RH, with a photoperiod of 16∶8 (L:D) h, and reared on artificial diet as described by Wyniger [27] and Bell and Joachim [28], respectively.

### Bacillus Thuringiensis Cry Proteins

A DNA fragment containing the *cry1A.105* gene was obtained from pEN08H-*cry1A.105* plasmid (provided by Bayer CropScience). The fragment was ligated into pGA64 plasmid (provided by Bayer CropScience) and used to transform *Escherichia coli* WK6 cells. Cry1Aa, Cry1Ab, Cry1Fa were expressed as protoxins in the recombinant *E. coli* strain WK6. Inclusion bodies purification and solubilization, protoxin activation by trypsin and toxin quantification were performed as described by Herrero *et al.* [29]. Cry1Ac was obtained from the *B. thuringiensis* strain HD73 (*Bacillus* Genetic Stock Collection, Columbus, OH), whereas Cry2Ab and Cry2Ae were obtained from the recombinant *B. thuringiensis* strains BtIPS78/11 and Bt1715 Cry^−^ mutant (Institut Pasteur, Paris) harbouring plasmid pGA32 expressing Cry2Ab and Cry2Ae, respectively. Crystal purification, solubilization and protoxin activation by trypsin were performed as described by Hernández-Rodríguez *et al.* [18].

The purity of the activated toxins was checked by 12% sodium dodecylsulfate polyacrylamide gel electrophoresis (12% SDS-PAGE). For all of them, a main fragment corresponding to the activated toxin was obtained. The activated toxins were kept at −20°C until used. For biochemical analyses, the activated proteins were further dialyzed in 20 mM Tris-HCl, pH 8.6 and filtered prior to anion-exchange purification in a MonoQ 5/5 column using an ÄKTA chromatography system (GE Healthcare, United Kingdom).

### Bioassays

Susceptibility to Cry toxins was tested with neonate larvae (<24 h old). Assays were conducted by the surface contamination method [30] using 128-cell trays (Bio-Ba-128, Color-Dec Italy, Frenchtown, NJ). Seven different concentrations of each toxin and a control with distilled water were tested using 16 larvae for each concentration. All assays were repeated 3–4 times for the active toxins and 2 times for the toxins with lower activity such as Cry2Ab and Cry2Ae. Mortality was assessed after 7 days. LC_50_ values were estimated from mortality data by Probit analysis [31] using the POLO-PC program (LeOra Software, Berkeley, CA). LC_50_ values were considered significantly different if their 95% fiducial limits (FL_95_) did not overlap.

### ^125^I Labeling of Cry Proteins

Trypsin-activated and chromatografically purified Cry1A.105, Cry1Fa, and Cry1Ab proteins were labeled with ^125^I (PerkinElmer, Boston, MA) using the chloramine T method [32], [33], (Fig. S1). Cry1A.105 and Cry1Fa (25 µg each) were labeled with 0.5 mCi of ^125^I, and Cry1Ab (25 µg) with 0.3 mCi. The specific activity of labeled proteins was 5.6, 0.5, and 1.2 mCi/mg, respectively.

### Brush Border Membrane Vesicles (BBMV) Preparation

BBMV from *O. nubilalis* and *S. frugiperda* were prepared by the differential magnesium precipitation method from dissected midguts of last instar larvae [34]. Protein concentration in the BBMV preparations was determined by the method of Bradford using BSA as standard [35].

### Binding Assays with ^125^I-labeled Cry Proteins

Prior to use, BBMV were centrifuged for 10 min at 16000×*g* and suspended in binding buffer (phosphate buffered saline (PBS), 0.1% BSA). To determine the optimal concentration of BBMVs for use in competition experiments, increasing amounts of BBMVs were incubated with either 0.3 nM of labeled-Cry1A.105, 0.4–1.0 nM of labeled-Cry1Ab, or 6.0 nM of labeled-Cry1Fa, in a final volume of 0.1 ml of binding buffer for 1 h at 25°C. An excess of unlabeled toxin (0.3 µM) was used to calculate the nonspecific binding. Homologous and heterologous competition experiments were performed in binding buffer incubating *O. nubilalis* or *S. frugiperda* BBMV with ^125^I-Cry1A.105, ^125^I-Cry1Ab, or ^125^I-Cry1Fa and increasing amounts of unlabeled toxins in a final volume of 100 µl for 1 h at 25°C. After incubation, samples were centrifuged at 16000×*g* for 10 min, and the pellets were washed with 500 µl of ice-cold binding buffer. Radioactivity in the pellets was measured in a model 1282 Compugamma CS gamma counter (LKB Pharmacia). Each competition experiment was conducted with duplicate points and was repeated a minimum of two times (see details in figure legends) with the same batch of BBMV, except for Cry1A.105 and *O. nubilalis*, for which two different BBMV preparations were used. Equilibrium dissociation constants (*K*_d_) and concentration of binding sites (*R*_t_) were estimated using the LIGAND program [36].

## Results

### Expression and Purification of the Cry1A.105 Protein

SDS-PAGE analysis of the induced *E. coli* culture showed a major protein band of the expected size (approx. 135 kDa) (data not shown). The protein was separated from contaminant *E. coli* proteins by anion-exchange chromatography (Fig. S2). SDS-PAGE of the chromatographic fractions showed that Cry1A.105 eluted in a sharp peak and essentially free of contaminant proteins (Fig. S2).

### Susceptibility of *O. nubilalis* and *S. frugiperda* to Cry Proteins

The results obtained in quantitative bioassays with Cry proteins are shown in Table 1. For *O. nubilalis*, all four Cry1 proteins were much more toxic than the two Cry2A proteins (at least 10-fold). Cry1A.105 was the most toxic one, with a difference in LC_50_ of around 10-fold compared to Cry1Ab, Cry1Ac, and Cry1Fa, which were equally toxic among them. The Cry2Ae protein was the least toxic for this insect.

**Table 1:** Toxicity of *Bacillus thuringiensis* Cry proteins to neonate larvae of *Ostrinia nubilalis* and *Spodoptera frugiperda* (measured after 5 days).

	LC_50_ (FL_95%_)^1^	
	*O. nubilalis*	*S. frugiperda*
**Cry1A.105**	0.6 (0.3–1)	400 (261–652)
**Cry1Ab**	6 (4–8)	783 (394–2282)
**Cry1Ac**	7 (5–10)	>4050^2^
**Cry1Fa**	5 (3–7)	35 (14–76)
**Cry2Ab**	85 (59–117)	>1350^2^
**Cry2Ae**	253 (151–388)	>1350^2^

1 LC_50_, 50% lethal concentration; FL, fiducial limits at the 95% level. Concentrations are expressed as ng/cm^2^.

2 The highest concentration used in the bioassay which produced less than 50% mortality.

Regarding *S. frugiperda*, Cry1Fa was the most active protein, followed by Cry1A.105 and Cry1Ab (equally toxic), which were more than 10-fold less active (Table 1). Cry1Ac, Cry2Ab and Cry2Ae were so little effective that LC_50_ values could not be obtained.

### Binding of ^125^I-labeled Cry1A.105 to BBMV

**Figure 1:**
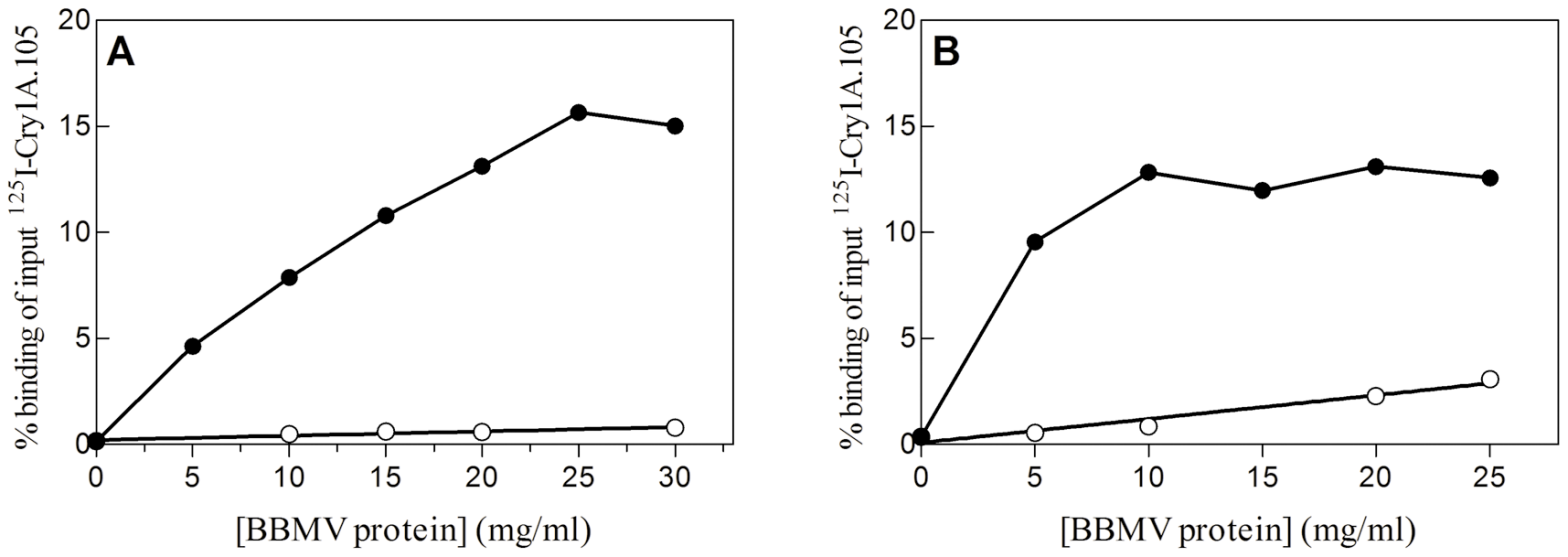
Binding of ^125^I-Cry1A.105 at increasing concentrations of BBMV proteins. (A) *O. nubilalis*; (B) *S. frugiperda*. •, Total binding; ○, non-specific binding.

**Figure 2:**
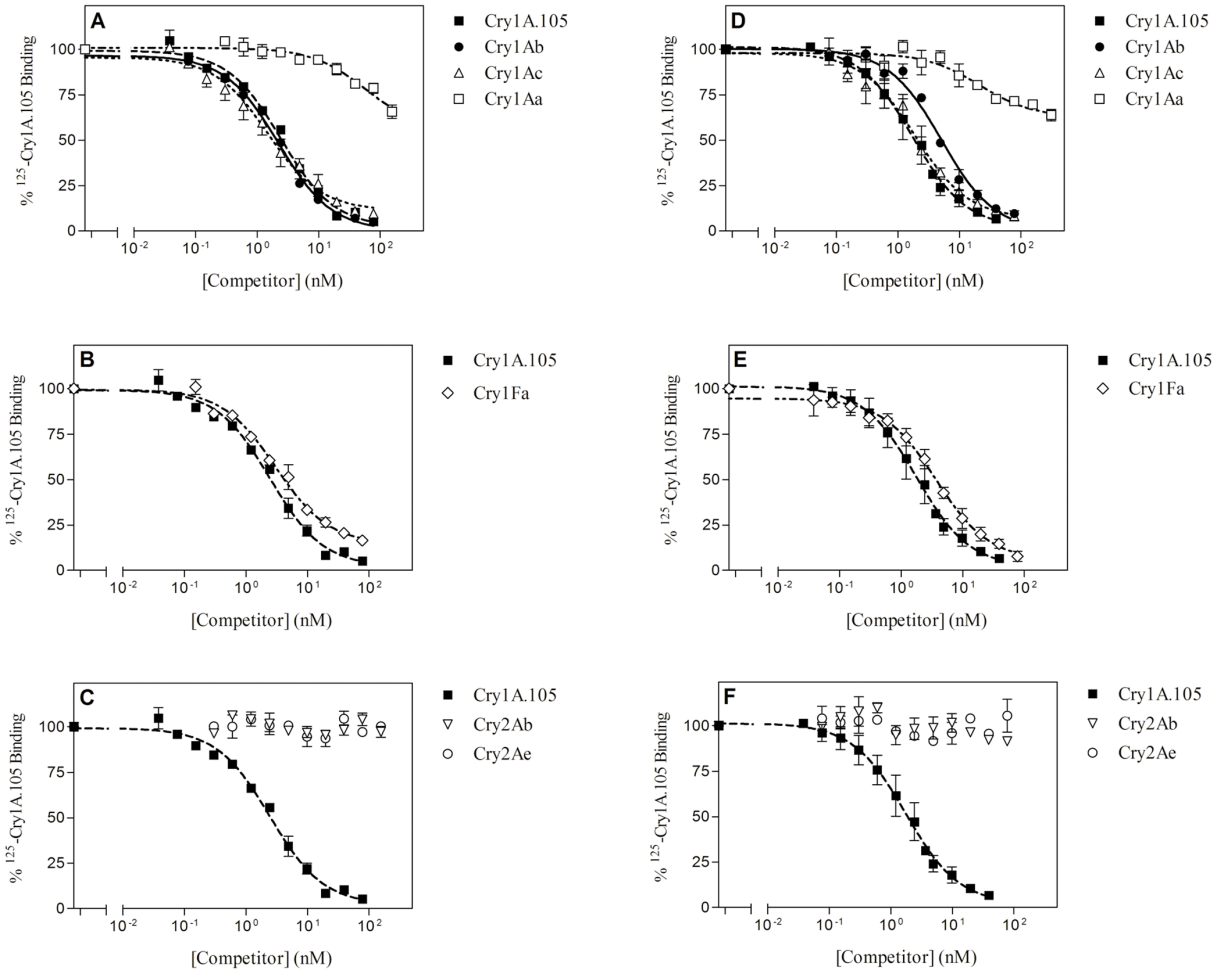
Competition binding experiments with ^125^I-Cry1A.105. Curves represent total binding of ^125^I-Cry1A.105 at increasing concentrations of unlabeled competitor, using BBMV from *O. nubilalis* (A, B, and C) or from *S. frugiperda* (D, E, and F). Each competition experiment (data points in duplicate) was replicated two to six times (competition with Cry1Fa was replicated six times in *S. frugiperda*; with Cry1A.105, five times in *S. frugiperda*; with Cry1Ab, four times in both species, and with Cry1Ac, three times in *O. nubilalis* and four times in *S. frugiperda*) and the error bars represent the standard error of the mean.

Specific binding of Cry1A.105 to BBMV from the two insect species is shown in Fig. 1. Approximately 12–15% of the labeled protein bound to BBMV. Dissociation constants (*K_d_*) and concentration of binding sites (*R_t_*) were estimated from the homologous competition (when the labeled protein and the competitor protein are the same) (Fig. 2). In both species, the values indicate that binding of Cry1A.105 is of high affinity, similar to those of other Cry1A proteins (Table 2).

**Table 2:** Binding parameters in Ostrinia nubilalis and Spodoptera frugiperda.

	*O. nubilalis* (Mean ± SEM)			*S. frugiperda* (Mean ± SEM)		
	*K_d_* (nM)	*R_t_* (pmol/mg)	*R_t/_K_d_*	*K_d_* (nM)	*R_t_* (pmol/mg)	*R_t/_K_d_*
**Cry1A.105**	1.9±0.2	1.28±0.11	0.7	1.56±0.15	2.6±0.2	1.7
**Cry1Ab**	0.18±0.03	0.48±0.04	2.7	0.33±0.09	0.88±0.09	2.7
**Cry1Fa**	0.5±0.2	0.40±0.03	0.8	2.5±0.4	0.61±0.05	0.2

Competition binding assays between labeled Cry1A.105 and the other Cry proteins are shown in Fig. 2. A similar pattern can be observed for the two insect species regarding Cry1A and Cry2A proteins: Cry1Ab and Cry1Ac compete completely for the Cry1A.105 binding sites, Cry1Aa only competes partially, and Cry2A proteins do not compete at all. A slightly different pattern was observed for competition by Cry1Fa, since this protein seems to not completely compete for all binding sites of Cry1A.105 in *O. nubilalis*, though it does in *S. frugiperda*.

### Binding of ^125^I-labeled Cry1Ab to BBMV

**Figure 3:**
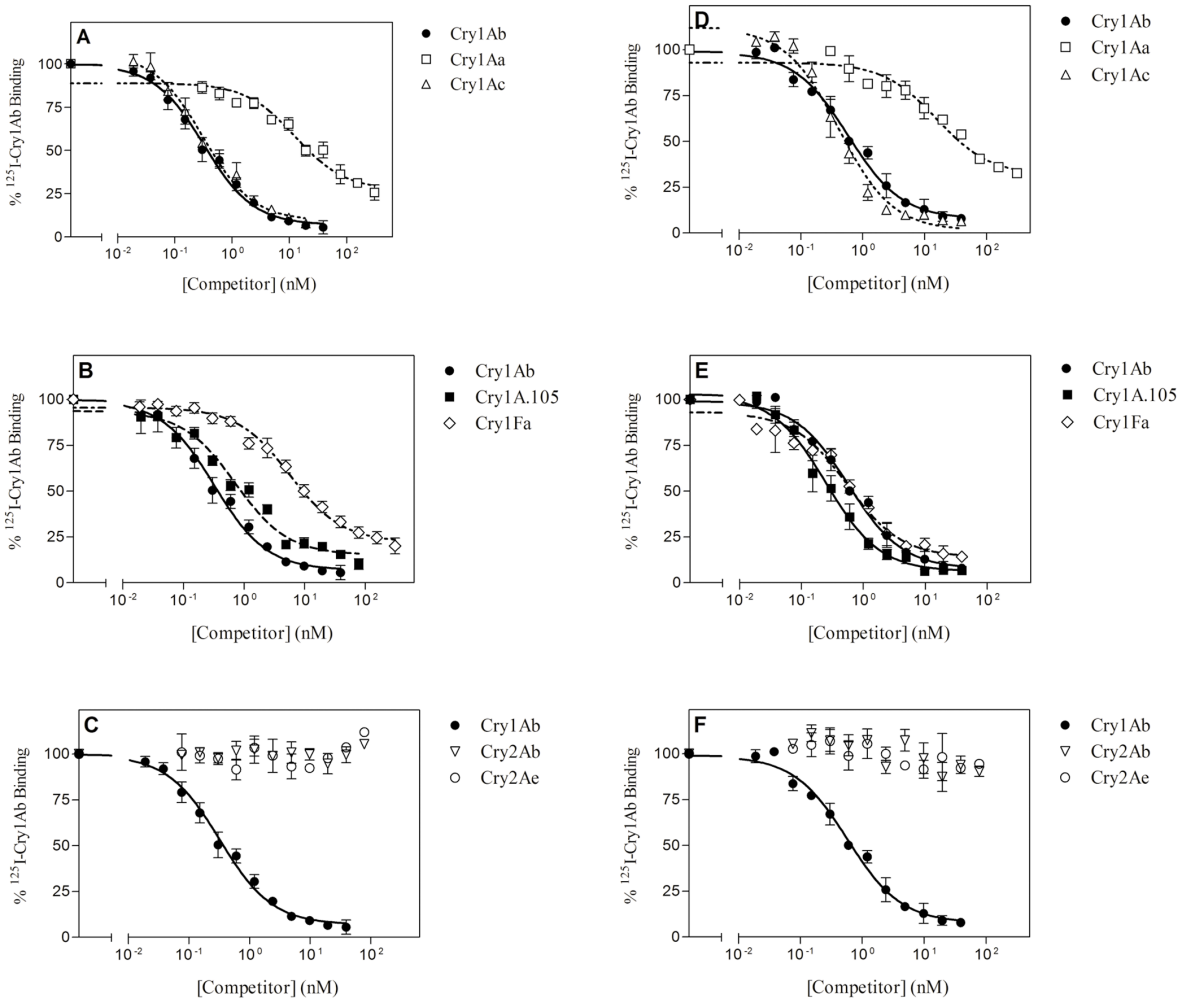
Competition binding experiments with ^125^I-Cry1Ab. Curves represent total binding of ^125^I-Cry1Ab at increasing concentrations of unlabeled competitor, using BBMV from *O. nubilalis* (A, B, and C) or from *S. frugiperda* (D, E, and F). Each competition experiment (data points in duplicate) was replicated two to eight times (competition with Cry1Fa was replicated eight times in *O. nubilalis* and 3 times in *S. frugiperda*; with Cry1Ab, five times in *O. nubilalis* and three times in *S. frugiperda*; with Cry1A.105, three times in *O. nubilalis* and five times in *S. frugiperda*, and with Cry1Ac and Cry2Ab, three times in both species) and the error bars represent the standard error of the mean.

Competition binding assays using labeled Cry1Ab are shown in Fig. 3. Again, a similar pattern can be observed for the two insect species regarding Cry1A and Cry2A proteins: Cry1Ac competes completely for the Cry1Ab binding sites, Cry1Aa only competes partially, and Cry2A proteins do not compete at all. Also, Cry1Fa and Cry1A.105 completely compete for Cry1Ab binding sites in *S. frugiperda*. However, in *O. nubilalis*, both Cry1Fa and Cry1A.105 seem to not compete completely for all Cry1Ab binding sites.

### Binding of ^125^I-labeled Cry1Fa to BBMV

**Figure 4:**
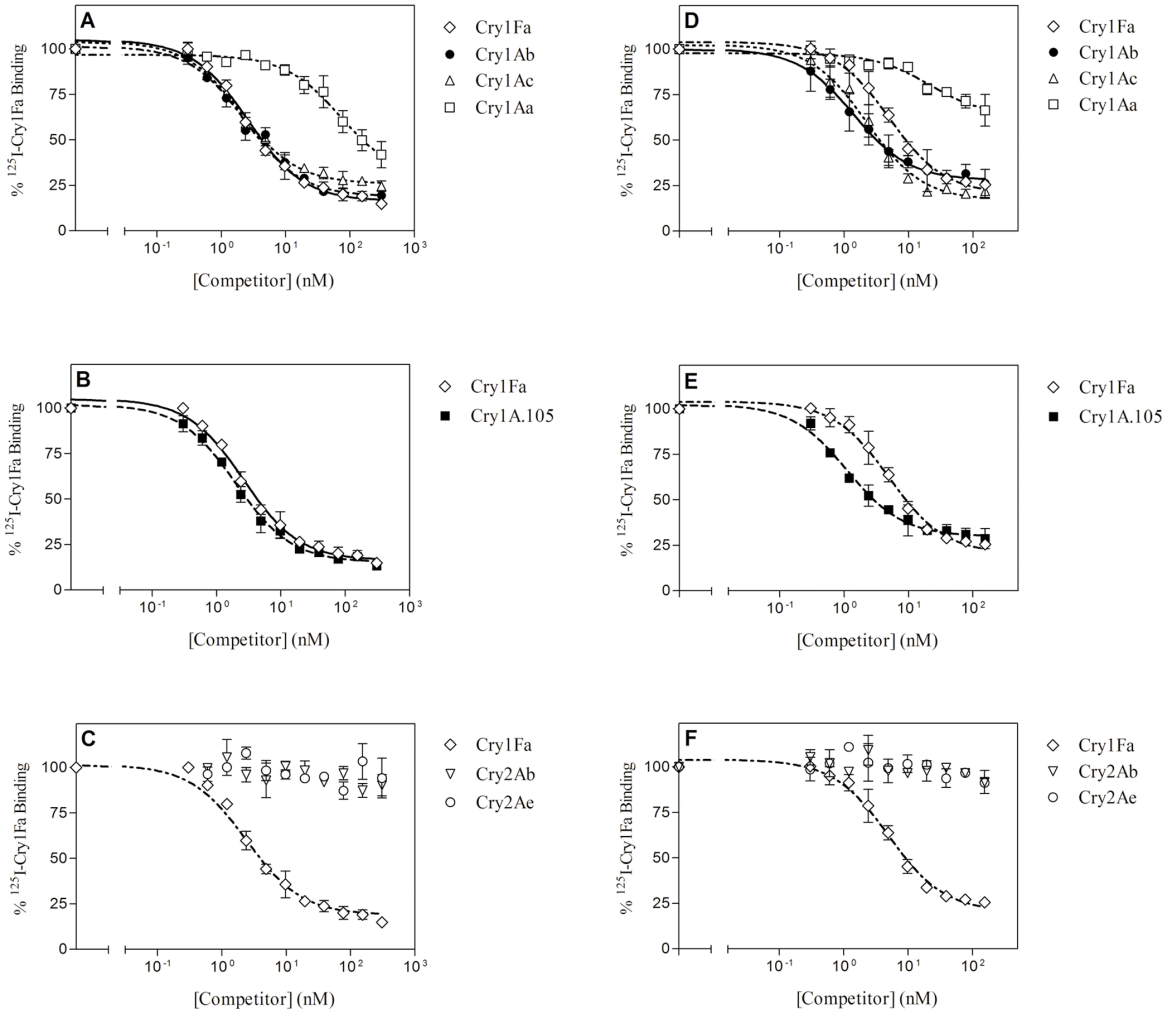
Competition binding experiments with ^125^I-Cry1Fa. Curves represent total binding of ^125^I-Cry1Fa at increasing concentrations of unlabeled competitor, using BBMV from *O. nubilalis* (A, B, and C) or from *S. frugiperda* (D, E, and F). Each competition experiment (data points in duplicate) was replicated two to three times in *O. nubilalis* (competition with Cry1Ab and Cry1Ac in *O. nubilalis* was replicated three times) and two times in *S. frugiperda* and the error bars represent the standard error of the mean.

Competition binding assays using labeled Cry1Fa are shown in Fig. 4. As with the other labeled proteins, similar results were obtained in both insect species regarding Cry1A and Cry2A proteins: Cry1Ab and Cry1Ac compete completely for the Cry1Fa binding sites, Cry1Aa only competes partially, and Cry2A proteins do not compete at all. Cry1A.105 completely competes for Cry1Fa binding sites in both species.

## Discussion

*Bacillus thuringiensis* is a good source of insecticidal proteins (over 250 have been described) (http://www.lifesci.sussex.ac.uk/home/Neil_Crickmore/Bt/, accessed 2013 Jun). Studies on the phylogenetic relationships of Cry proteins have led to hypothesize that natural Cry proteins have evolved by sequence divergence and by domain swapping through homologous recombination [9], [37]. Despite this huge diversity, the number of active proteins for the control of any given pest can be very limited, depending on the species [38]. Domain swapping was explored in the mid 90′s to create new combinations among the existing domains of natural proteins to generate chimeric Cry proteins with broader spectrum of activity or with increased toxicity [39], [40].

Several aspects of the mode of action of Cry proteins are still under debate [41], but the initial specific binding step is common in all models proposed and it is considered critical for toxicity. Studies on the roles of Cry protein domains has evidenced that domain I is involved in the insertion into the epithelial membrane, and domains II and III are related with the interaction with binding sites in the midgut epithelium [29], [42]–[45]. Competition binding experiments have provided models for binding sites to predict or to explain patterns of cross-resistance or multiple resistance [13]–[16], [24], [46]. In most insect species tested so far, competition of Cry1Fa for Cry1Ab or Cry1Ac binding sites has been found, although, in general, with low affinity [23]–[25], [47]. This has led to the proposal that Cry1Fa might have binding sites not shared with those of Cry1A proteins, in addition to the one shared with low affinity. The functional involvement of the shared binding site in the toxicity pathway is corroborated, at least, by two cases of strong cross-resistance to Cry1Fa in insects selected with Cry1A proteins: one in *Plutella xylostella* [22] and the other in *H. virescens* [20]. In both cases, Cry1A proteins and Cry1Fa were shown to share a common binding site [23], [24]. Binding analysis of the resistant *P. xylostella* insects indicated extremely reduced binding of Cry1A proteins (Cry1Ab and Cry1Ac, and one of the two sites of Cry1Aa) [48] and Cry1Fa [17]. In the resistant *H. virescens* insects, binding of the three CryA proteins and Cry1Fa was absent [49]. Furthermore, genetic evidence in resistant *P. xylostella* indicated that a single gene conferred resistance to Cry1A proteins and Cry1Fa [50]. These results provide an example that an alteration of a shared step in the mode of action (in this case, binding to a common site) is sufficient to confer resistance to Cry1A and Cry1Fa proteins.

The results obtained in the present study indicate that the chimeric protein Cry1A.105 is more efficient to control *O. nubilalis* than the two parental proteins (Cry1Ab and Cry1Fa). For control of *S. frugiperda*, it is not significantly more active than Cry1Ab and around 10-fold less active than Cry1Fa, at least in our assay conditions and with our laboratory insect strains (Table 1). Regarding binding sites, both *O. nubilalis* and *S. frugiperda* have common binding sites of high affinity for Cry1Ab, Cry1Ac, Cry1A.105 and Cry1Fa, which are not shared by Cry2Ab and Cry2Ae proteins. Binding parameters of Cry1A.105 and Cry1Ab are similar in the two species, although the toxicity profiles differ between both insects. This observation may indicate the involvement in toxicity of other steps in the mode of action unrelated to binding. The low efficiency of Cry1Aa competing for Cry1A.105 and Cry1Ab binding sites may shed light on the type of protein domains interacting with the shared site. Cry1Ab can be considered a natural chimera between Cry1Aa (domain III) and Cry1Ac (domains I and II): Cry1Ab and Cry1Ac share a high degree of amino acid similarity in domain II and Cry1Aa is more divergent, whereas Cry1Aa and Cry1Ab are very similar in domain III, but not Cry1Ac [9], [37], [51]. Thus, the low affinity of Cry1Aa, along with the high affinity of Cry1Ac for the shared binding site, could be interpreted as a strong indication of the importance of domain II in the binding of these proteins to the shared site.

Our binding site model is supported by some studies that showed the occurrence of shared binding sites for Cry1Ab and Cry1Ac in *O. nubilalis* [52]–[54], as well as the occurrence of shared binding sites in *S. frugiperda* for Cry1Ab and Cry1Ac [55], and for either Cry1Ab or Cry1Ac and Cry1Fa [26], [47]. Hua *et al*. [54] reported contradictory data for reciprocal competition between Cry1Ab and Cry1Fa in *O. nubilalis* using surface plasmon resonance (with immobilized Cry protein and BBMV preincubated with the competitor Cry protein). Using radiolabeled toxins, they found competition of Cry1Fa with radiolabeled Cry1Ab, though only at very high concentrations of competitor (50% inhibition at 10,000-fold). In our study we obtained 50% binding inhibition of radiolabeled Cry1Ab at 20-fold Cry1Fa. It is possible that differences between both studies in the preparation of BBMV or the Cry1Fa protein could affect recognition of Cry1Fa to the Cry1Ab binding site.

Several populations of *O. nubilalis* have been selected in the laboratory for resistance to Cry1 proteins [2]. In the few cases where cross-resistance among Cry1Ab or Cry1Fa has been tested, little or no cross-resistance has been found. Two independently derived populations with moderate (35- to 39-fold) or high (>535-fold) resistance to Cry1Ab and Cry1Ac showed essentially no cross-resistance to Cry1Fa (2-fold and 6-fold, respectively) [56], [57]. Likewise, a colony highly resistant to Cry1Fa (>3000-fold) had negligible cross-resistance to Cry1Ac and no cross-resistance to Cry1Ab [58]. In the colonies selected for resistance to Cry1Ab, binding of Cry1Ab or Cry1Ac was not affected; only binding of Cry1Aa was decreased [56], [57]. A reduction in the amount of cadherin in BBMV was detected in one of the resistant colonies [59]. In the colony selected for Cry1Fa resistance, binding of this toxin to BBMV was not affected [60] and the gene responsible of resistance was mapped to a chromosome different from those carrying known Cry1A receptor genes (cadherin, alkaline phosphatase, and aminopeptidase) [61]. The limited cross-resistance pattern observed in this species is indicative of a very specific mechanism, such as binding site alteration. We cannot exclude the possibility that the evidence for binding alteration has remained elusive so far due to the occurrence of futile binding to other sites that masks any alteration of the site responsible for the toxicity. Furthermore, since the number of *O. nubilalis* resistant populations tested is small and resistance has always been obtained under laboratory conditions, the possibility of finding evidence for receptor modification as the basis for field-evolved resistance in *O nubilalis* cannot be discarded.

A resistant strain of *Diatraea saccharalis*, selected with Cry1Ab up to a resistance level of >100-fold, was tested for cross-resistance to Cry1A.105 as well as to other Cry1A and Cry2A proteins [62]. The data showed contradictory results depending on whether mortality or “practical mortality” (dead larvae plus surviving larvae with a body weight <0.1 mg) was considered. Thus, considering “practical mortality”, the levels of cross-resistance were 4.1-, 45-, –>80-, and –0.5-fold, to Cry1A.105, Cry1Ac, Cry1Aa, and Cry2Ab, respectively. However, considering actual mortality, resistant insects showed high levels of cross-resistance to Cry1A.105 (>40-fold), low levels to Cry1Ac (around 10-fold), and no cross-resistance to either Cry1Aa or Cry2Ab. These results show that cross-resistance to Cry1A.105 is possible in insects that have been selected with Cry1Ab.

There are no reports of laboratory selection of *S. frugiperda* with Cry proteins. However, this species has developed resistance in the field to Bt-corn expressing Cry1Fa [63], [64]. Laboratory bioassays with individual toxins resulted in a level of resistance of >311-fold for Cry1Fa, whereas only moderate resistance to Cry1Ab (22-fold) and to Cry1Ac (35-fold). Binding assays showed that resistant insects lack binding of Cry1Ab, Cry1Ac, and Cry1Fa proteins [65].

In summary, our results indicate that Cry1A.105 can be an alternative to Cry1Ab/Ac for the control of *O. nubilalis*, but it appears inferior to Cry1Fa for the control of *S. frugiperda.* Based on the results on binding site interactions, the development of cross-resistance among Cry1Ab/Ac proteins, Cry1A.105, and Cry1Fa appears possible in *O. nubilalis* and *S. frugiperda*, since a mutation altering a shared binding site could occur. Conversely, cross-resistance between these proteins and Cry2A proteins is very unlikely in such case. The current study supports the importance of the establishment of binding models for Cry proteins as an essential tool during the design of effective pyramided Bt-crops.

## Supporting Information

Figure S1 **Autoradiography of the ^125^I-labeled Cry proteins.** Only the two first fractions eluting from the desalting column are shown. (A) Cry1A.105, (B) Cry1Ab, and (C) Cry1Fa. Arrows indicate the position of the Cry protein.
(TIF)

Figure S2 **Purification of Cry1A.105 by anion-exchange chromatography.** (A) Chromatogram indicating the start and end of the injection (broken vertical lines), the linear gradient of 1 M NaCl (inclined line) and the absorbance profile at 280 nm; the peak corresponding to Cry1A.105 is marked with an arrow. (B) SDS-PAGE with Coomassie blue staining of some of the fractions; M, molecular mass marker; B10–D6, fraction number.
(TIF)
