# Supplementary figures and images for: Shared Midgut Binding Sites for Cry1A.105, Cry1Aa, Cry1Ab, Cry1Ac and Cry1Fa Proteins from Bacillus thuringiensis in Two Important Corn Pests, Ostrinia nubilalis and Spodoptera frugiperda
Source: PLoS One. 2013 Jul 5;8(7):e68164. doi: 10.1371/journal.pone.0068164 (PMC3702569; doi:10.1371/journal.pone.0068164)

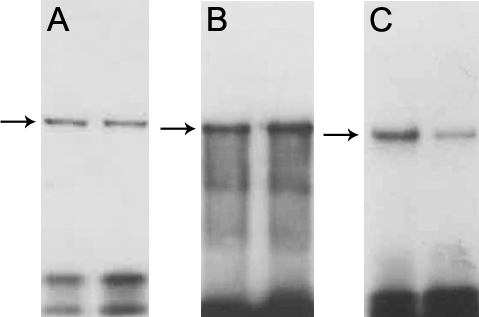

Supplement: Figure S1 — Autoradiography of the 125I-labeled Cry proteins. Only the two first fractions eluting from the desalting column are shown. (A) Cry1A.105, (B) Cry1Ab, and (C) Cry1Fa. Arrows indicate the position of the Cry protein. (TIF) [file pone.0068164.s001.tif]

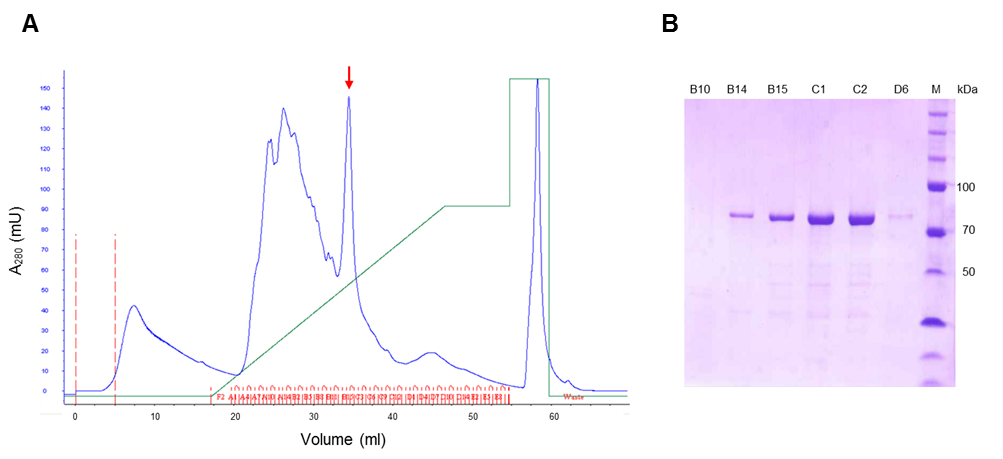

Supplement: Figure S2 — Purification of Cry1A.105 by anion-exchange chromatography. (A) Chromatogram indicating the start and end of the injection (broken vertical lines), the linear gradient of 1 M NaCl (inclined line) and the absorbance profile at 280 nm; the peak corresponding to Cry1A.105 is marked with an arrow. (B) SDS-PAGE with Coomassie blue staining of some of the fractions; M, molecular mass marker; B10–D6, fraction number. (TIF) [file pone.0068164.s002.tif]
